# Diagnostic accuracy of artificial intelligence models in detecting congenital heart disease in the second-trimester fetus through prenatal cardiac screening: a systematic review and meta-analysis

**DOI:** 10.3389/fcvm.2025.1473544

**Published:** 2025-02-24

**Authors:** Lies Dina Liastuti, Yosilia Nursakina

**Affiliations:** ^1^ Department of Cardiology and Vascular Medicine, Faculty of Medicine Universitas Indonesia, Cipto Mangunkusumo Hospital, Jakarta, Indonesia; ^2^ Department of Cardiovascular, Harapan Kita National Heart Center, Jakarta, Indonesia; ^3^ School of Public Health, Imperial College London, London, United Kingdom

**Keywords:** artificial intelligence, congenital heart disease, meta-analysis, prenatal cardiac examination, ultrasonography

## Abstract

**Background:**

Congenital heart disease (CHD) is a major contributor to morbidity and infant mortality and imposes the highest burden on global healthcare costs. Early diagnosis and prompt treatment of CHD contribute to enhanced neonatal outcomes and survival rates; however, there is a shortage of proficient examiners in remote regions. Artificial intelligence (AI)-powered ultrasound provides a potential solution to improve the diagnostic accuracy of fetal CHD screening.

**Methods:**

A literature search was conducted across seven databases for systematic review. Articles were retrieved based on PRISMA Flow 2020 and inclusion and exclusion criteria. Eligible diagnostic data were further meta-analyzed, and the risk of bias was tested using Quality Assessment of Diagnostic Accuracy Studies—Artificial Intelligence.

**Findings:**

A total of 374 studies were screened for eligibility, but only 9 studies were included. Most studies utilized deep learning models using either ultrasound or echocardiographic images. Overall, the AI models performed exceptionally well in accurately identifying normal and abnormal ultrasound images. A meta-analysis of these nine studies on CHD diagnosis resulted in a pooled sensitivity of 0.89 (0.81–0.94), a specificity of 0.91 (0.87–0.94), and an area under the curve of 0.952 using a random-effects model.

**Conclusion:**

Although several limitations must be addressed before AI models can be implemented in clinical practice, AI has shown promising results in CHD diagnosis. Nevertheless, prospective studies with bigger datasets and more inclusive populations are needed to compare AI algorithms to conventional methods.

**Systematic Review Registration:**

https://www.crd.york.ac.uk/prospero/display_record.php?ID=CRD42023461738, PROSPERO (CRD42023461738).

## Introduction

Congenital heart disease (CHD) is the most common congenital abnormality, affecting approximately 1% of live births worldwide (1). All CHD cases require life-long follow-up (2), with around one in four requiring at least one cardiac surgery within their first year of life (3). Thus, CHD contributes significantly to morbidity and infant mortality (4) and imposes the highest burden on global healthcare costs (5). While the incidence of CHD is comparable across the globe, the weight of this burden is particularly pronounced in low- and middle-income countries (LMICs), especially those characterized by high fertility rates, such as Indonesia (6, 7). It has been determined that early diagnosis and prompt treatment of CHD, like prenatal cardiac examination, contribute to enhanced neonatal outcomes and survival rates (8). It is recommended that cardiac screening be performed between 18 and 22 weeks of gestation using a general obstetric ultrasound with a specified ultrasound probe for a focused evaluation of fetal heart (9–11).

CHD screening in newborns exhibits a moderate sensitivity of 68.5% and a high rate of false negatives, which may lead to delayed diagnosis and adverse events (12). This could be attributed to artifacts, making it challenging to identify small details and structures (13). Current data indicate that CHD detection rates remain low, at just 48%, particularly in low- and middle-income regions, possibly due to the shortage of skilled examiners in rural and remote areas (14). The accuracy of ultrasound results highly depends on the proficiency of examiners, which is influenced by technique, knowledge, and experience (15).

To bridge the gap between the high demand for prenatal screening for CHD and limited resources, integrating artificial intelligence (AI) presents a promising solution. AI involves leveraging machines and systems to imitate human problem-solving and decision-making capabilities. One type of AI, machine learning (ML), utilizes algorithms to identify patterns and predict outcomes from predetermined data. Deep learning (DL), a subset of ML, is an unsupervised AI technique that consistently outperforms traditional ML methods and can organize data into multiple processing layers, enabling autonomous learning, aiding decision-making, and revealing new findings that may otherwise elude human detection (12–14).

Numerous studies have shown that AI holds great promise in the early detection of CHD by distinguishing various cardiac abnormalities (16), enhancing the quality of ultrasound images (17, 18), streamlining the segmentation of cardiac structures (19, 20), assisting in ultrasound image acquisition (21, 22), and quantifying echocardiographic measurements (23, 24). The integration of AI with fetal ultrasound has been shown to significantly improve clinical efficiency, reduce subjective variability due to operator expertise differences, standardize plane acquisition, and provide potential solutions for areas with scarce medical resources (10, 13).

To date, no quantitative synthesis has been conducted on the application and accuracy of artificial intelligence models in detecting congenital heart disease through prenatal cardiac screening. This systematic review and meta-analysis aims to summarize recent research findings on AI's diagnostic performance in CHD diagnosis during the second trimester of pregnancy.

The paper is organized as follows: the *Methods* section outlines the search strategy, selection criteria, and statistical methods used in the systematic review and meta-analysis, including data extraction and quality assessment. The *Results* section presents the findings of the meta-analysis, including the diagnostic performance of AI models in CHD detection. This is followed by a detailed *Discussion* on the implications of AI integration in clinical practice, study heterogeneity, limitations, and potential future directions. Finally, the *Conclusion* section summarizes the key findings and emphasizes the potential of AI to improve CHD diagnosis, particularly in low-resource settings.

## Methods

### Search strategy and selection criteria

This review adhered to the Preferred Reporting Items for Systematic Reviews and Meta-Analyses (PRISMA) recommendations (25) and is registered with PROSPERO, number CRD42023461738. Seven databases, namely Embase, PubMed, MEDLINE, Cochrane, Global Health, IEEE Xplore, and Scopus, were systematically searched up to 30 September 2023. The reference lists of all relevant articles were also reviewed to enhance the identification of published AI research. Titles and abstracts were independently reviewed by one researcher, and all relevant citations were included for full-text analysis. Since this study only involved retrieving and synthesizing data from already published studies, ethical approval was not necessary. The complete search strategy adopted for each database is summarized in the Supplementary Material.

### Study eligibility

The Population, Intervention, Comparison, Outcome (PICO) search framework was applied in the screening and interpretation processes, as described below:

- Population: studies conducted on humans, limited to second-trimester fetuses (aged 13–26 weeks), the gold standard period for fetal organ (especially cardiac) screening through prenatal cardiac screening, regardless of geographical location.
- Intervention: prenatal ultrasound or echocardiography screening augmented with AI, including but not limited to machine learning and deep learning techniques.
- Comparator: clinician diagnosis of CHD based on the patient's medical examination results, including but not limited to patient interview, physical examinations, laboratory tests, and radiology imaging.
- Outcomes: the overall performance or accuracy parameters of artificial intelligence, which can include sensitivity, specificity, negative predictive value, positive predictive value (precision), F1 score, receiver operating characteristic (ROC) curve, area under the curve (AUC), and Dice coefficient.

The exclusion criteria were as follows: editorials, letters, reviews, conference proceedings, pre-prints, any articles in languages other than English, and any articles not related to the research topic.

### Data extraction and quality assessment

One reviewer independently extracted study characteristics and diagnostic outcomes using a standardized data extraction form. The recorded data from each study included authors’ names, publication year, AI methods, training and testing datasets, and results (including sensitivity, specificity, accuracy, F1 score, AUC). To identify any risk of bias, each study was appraised using the Quality Assessment of Diagnostic Accuracy Studies—Artificial Intelligence (QUADAS-AI), a framework designed to evaluate the risk of bias and applicability in reviews of AI diagnostic test accuracy and comparative accuracy studies that use at least one AI-centered index test. Three domains were assessed for risk of bias and concerns regarding applicability: patient selection, index test, and reference standard. The patient selection domain was additionally assessed based on the flow and timing of the study. If all domains related to bias or applicability in a study are deemed “low,” it is acceptable to give an overall judgment of “low risk of bias” or “low concern regarding applicability.” However, if a study is deemed “high” or “unclear” on one or more domains, it may be considered “at risk of bias” or have “concerns regarding applicability” (26).

### Statistical analysis

The true positives, false positives, true negatives, and false negatives were pooled to generate sensitivity and specificity for CHD diagnosis. A meta-analysis, performed using the R package meta, was used to construct forest plots for sensitivity and specificity using the inverse-variance model (27). Heterogeneity was assessed using Cochran's *Q*-test and the Higgins inconsistency index (*I*^2^) test. *P* <0.05 in Cochran's *Q*-test indicated the existence of heterogeneity, while a Higgins *I*^2^ test value >50% indicated substantial heterogeneity. As high heterogeneity between studies was suspected, a random-effects model was used for synthesis. Hierarchical summary receiver operating characteristics curves and 95% confidence intervals (CIs) were estimated using the Reitsma bivariate model (28) using R package mada (29). Deeks’ funnel plot of the asymmetry test was not possible due to the number of studies being fewer than 10. All statistical analyses were performed using R version 4.2.1 (R Statistical Computing).

## Results

**Figure 1:**
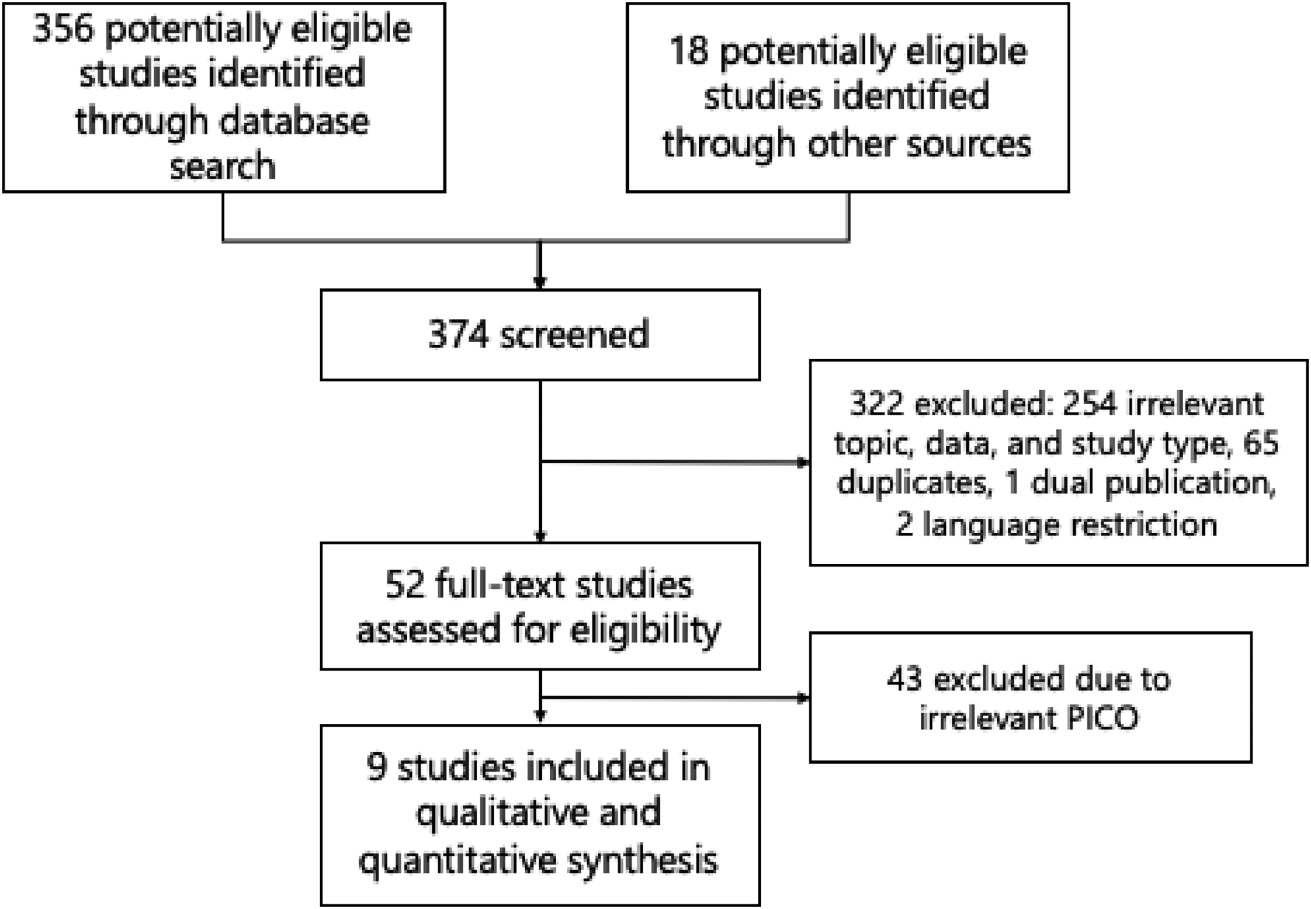
Flow diagram of the study selection.

A total of 374 studies were identified using the search strategy, as shown in the PRISMA flow diagram in Figure 1. After excluding duplicates and irrelevant articles, only 52 studies underwent a full-text review to assess eligibility. Ultimately, nine original articles with sufficient data to construct a 2 × 2 table were included in this review and meta-analysis (16, 30–37). The quality assessment results are displayed in Table 1, which suggests that most studies had a low risk of bias and low applicability concerns. The risk of bias in four studies (31–34) is mainly due to unclear patient selection methods or database sources and indefinite division between training and testing datasets.

**Table 1:** Summary of the risk of bias and applicability concerns.

No.	Study	Risk of bias				Applicability concerns		
		Patient selection	Index test	Reference standard	Flow and timing	Patient selection	Index test	Reference standard
1	Arnaout et al. (16)	Low	Low	Low	Low	Low	Low	Low
2	Gong et al. (30)	Low	Low	Low	Low	Low	Low	Low
3	Nurmaini et al. (31)	Low	Low	Low	Low	Low	Low	Low
4	Qiao et al. (32)	High	Low	Unclear	Unclear	Low	Low	Low
5	Tang et al. (33)	Unclear	Low	Low	Low	Low	Low	Low
6	Truong et al. (34)	High	Low	Low	Low	Low	Low	Low
7	Wang et al. (35)	Low	Low	Low	Low	Low	Low	Low
8	Wu et al. (36)	Low	Low	Low	Low	Low	Low	Low
9	Yang et al. (37)	Low	Low	Low	Low	Low	Low	Low

Among nine studies in Table 2, only one used ML instead of DL for diagnosing CHD (34). Half of the included studies used ultrasound images (16, 31, 32, 36, 37), whereas the others analyzed echocardiography images. All studies described and divided the training and testing datasets used in their study, except for two studies (32, 34). The number of videos in the training and testing datasets ranges from as few as 50 to over 100,000 ultrasound images. However, most studies exhibit an imbalanced ratio, with more training data than testing data. This is likely due to the rarity of detecting CHD in prenatal cardiac screening. One study specifically examined total anomalous pulmonary venous connection (TAPVC) (35), while others distinguished CHDs in general from normal heart images. Only a few studies conducted external and cross-validation to ensure the reliability of their models prior to clinical deployment in real-world settings (16, 30, 33, 34). The AI models performed exceptionally well in accurately identifying normal and abnormal ultrasound images. They exhibited a sensitivity range of 68%–100%, specificity range of 84%–100%, accuracy range of 83%–100%, F1 score range of 66%–100%, and AUC range of 0.88–0.99.

**Table 2:** Summary of the studies included in the meta-analysis.

No.	Study	AI method	Training dataset	Testing dataset	Results
1	Arnaout et al. (16)	DL: convolutional neural network (CNN)	107,823 images	4,108 patients: 4,071 normal, 37 diseased	Sensitivity 88% (95% CI: 47%–100%); specificity 90% (95% CI: 73%–98%); accuracy 88%; F1 94%; AUC 0.92
2	Gong et al. (30)	DL: CNN	3,196 images (2,655 normal vs. 541 diseased)	400 patients: 200 normal, 200 diseased	Sensitivity 85% (95% CI: 79%–90%); specificity 90% (95% CI: 85%–94%); accuracy 88%; F1 87%; AUC 0.881
3	Nurmaini et al. (31)	DL: CNN	969 images (157 normal vs. 812 diseased)	160 patients: 20 normal, 140 diseased (intra-patient)	Sensitivity 100% (95% CI: 95%–100%); specificity 100% (95% CI: 71%–100%); accuracy 100%; F1 100%
4	Qiao et al. (32)	DL: CNN	50 ultrasound videos: 25 normal, 25 diseased	N/A	Sensitivity 94% (95% CI: 80%–100%); specificity 92% (95% CI: 74%–99%); accuracy 95%; F1 95%
5	Tang et al. (33)	DL: CNN	6,698 images	350 patients: 200 normal, 150 diseased	Sensitivity 97% (95% CI: 93%–99%); specificity 99% (95% CI: 96%–100%); accuracy 98%; F1 98%; AUC 0.996
6	Truong et al. (34)	ML: random forest	3,910 patients	N/A	Sensitivity 85% (95% CI: 82%–88%); specificity 88% (95% CI: 87%–89%); accuracy 88%; F1 66%; AUC 0.94
7	Wang et al. (35)	DL: CNN	540 videos	120 patients: 82 without TAPVC, 20 with TAPVC	Sensitivity 90% (95% CI: 67%–99%); specificity 87% (95% CI: 77%–93%); accuracy 88%; F1 72%; AUC 0.941
8	Wu et al. (36)	DL: CNN	1,395 images (800 normal vs. 595 diseased)	300 patients: 154 normal, 146 diseased	Sensitivity 97% (95% CI: 92%–99%); specificity 84% (95% CI: 78%–90%); accuracy 90%; F1 91%
9	Yang et al. (37)	DL: CNN	1,395 images	123 patients: 66 normal, 57 diseased	Sensitivity 68% (95% CI: 55%–80%); specificity 95% (95% CI: 87%–99%); accuracy 83%; F1 79%

**Figure 2:**
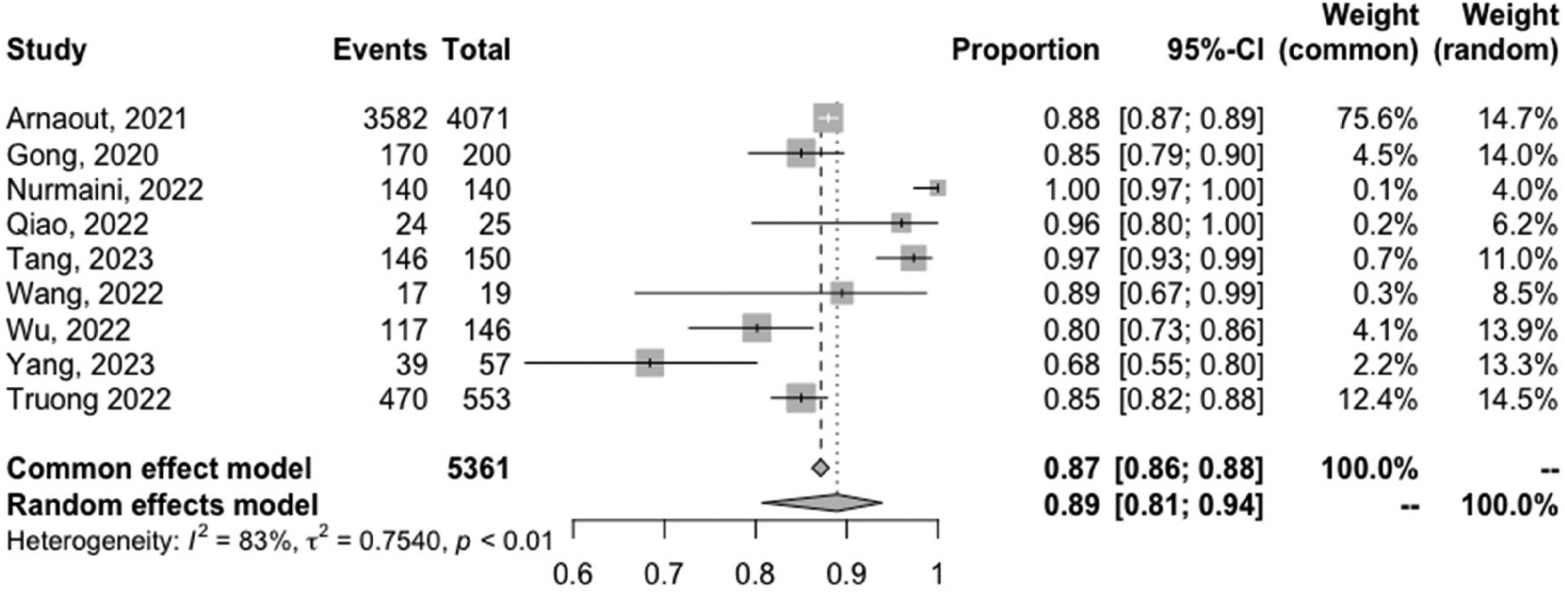
Forest plots of the pooled sensitivity for the diagnostic performance of AI in detecting CHD.

**Figure 3:**
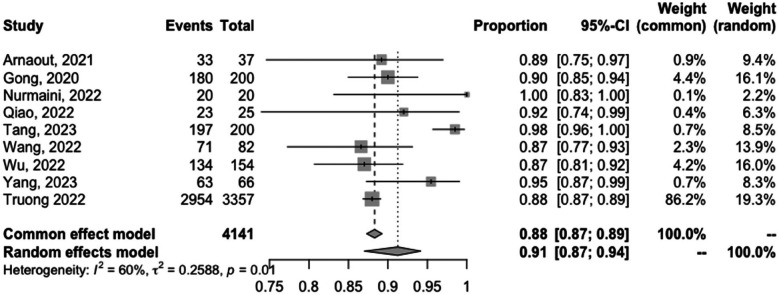
Forest plots of the pooled specificity for the diagnostic performance of AI in detecting CHD.

**Figure 4:**
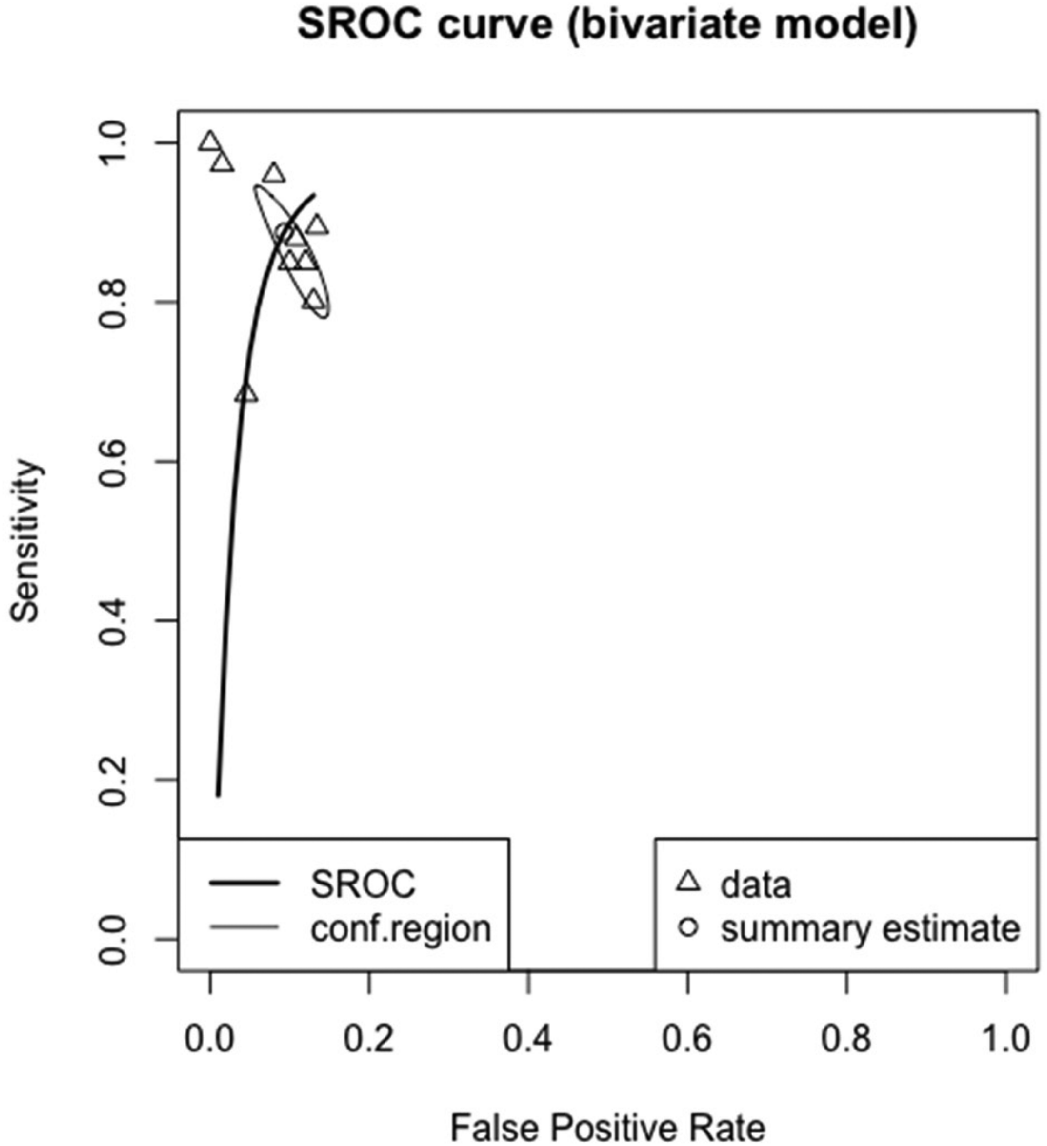
SROC curve for the diagnostic performance of AI in detecting CHD.

The meta-analyzed sensitivity and specificity of these nine studies are shown in Figures 2 and 3, respectively. The heterogeneity of all studies was high for both forest plots, with 83% for sensitivity and 60% for specificity; hence, random-effects quantity models were used for the meta-analysis. From the random-effect models, the overall sensitivity and specificity were 0.89 (0.81–0.94) and 0.91 (0.87–0.94), respectively. The summary receiver operating curve (SROC) was also plotted, as can be seen in Figure 4, with a pooled AUC of 0.952.

## Discussion

CHD remains the most prevalent congenital disability disease and is the leading cause of infant mortality (38). Improving the early diagnosis and screening rate of fetal CHD is crucial. Ultrasound is the most commonly used imaging modality and an essential tool in clinical practice due to its low cost, non-invasive nature, and high reproducibility (39). However, the quality of fetal echocardiographic images affects the assessment of cardiac structure, function, and prenatal diagnostic outcomes. Obtaining high-quality and standard fetal echocardiographic images remains challenging due to factors such as fetal position, differences in sonographer skill levels, and variations in instrument resolution. Diagnosis relies heavily on the sonographer's experience, leading to unsatisfactory detection rates for fetal cardiac abnormalities (40). Integrating AI into the diagnostic process for early detection of CHD is highly beneficial for reducing morbidity and mortality.

This systematic review and meta-analysis is the first to assess the effectiveness of AI in diagnosing CHDs during prenatal cardiac screening in second-trimester fetuses. The second trimester is specifically studied because it offers more reliable fetal orientation and better assessment of heart development (41). This review provides a more updated and thorough evaluation compared to the previous review on AI's use in CHD diagnosis using fetal echocardiography.

According to this study, AI models demonstrate very high performance in detecting CHD compared to conventional methods (i.e., clinician's diagnosis of CHD). The DenseNet 201 model, tested on an intra-patient dataset in a study by Qiao et al. (32), achieved 100% sensitivity and specificity and thus 100% accuracy. This could be achieved by combining gradient class activation mapping (Grad–CAM) with guided backpropagation (Guided-BP). Abnormal pixels in ultrasound images are highlighted and visualized, which improves the interpretability and understanding of expert fetal cardiologists.

Other than that, other AI models also demonstrated high diagnostic accuracy. For instance, OB-4000, used by Arnaout et al. (16), employed the biggest testing dataset, which is said to simulate the real prevalence of CHD in a typical population (0.8%–1%). Their work is the closest translation to resource-poor and real-world settings. Therefore, automatic screening for CHD through these AI algorithms might overcome the need for expert examiners and increase the CHD detection rate. On a population level, this will greatly assist both beginners and expert clinicians in diagnosing CHD as well as broaden access to fetal heart screening.

Wu et al. (36) further analyzed that AI can even provide high-quality teaching tools to aid sonographers in learning about CHD. While most studies focus on differentiating between normal and CHD hearts, classifying different types of CHD is very crucial for further treatment and knowing the prognosis, as done by Nurmaini et al. (31). However, as the number of classification classes increases, the accuracy, sensitivity, and specificity of AI algorithms decrease. They were able to increase the accuracy to as high as 99% by employing geometric transformation and increasing the training dataset, which is very crucial in a deep learning AI model. Having more robust and efficient AI algorithms is also the key to translating into resource-poor and real-world settings.

AI models have shown high accuracy in detecting CHD, which suggests that integrating AI into routine prenatal cardiac screening could potentially reduce healthcare costs, especially in LMICs. Although no studies have specifically examined the cost-effectiveness of AI-augmented prenatal cardiac screening, one study found that AI-augmented ECG examination could be the most cost-effective option, with a cost of less than $50,000 per quality-adjusted life year (QALY) willingness-to-pay threshold (42).

While machine learning algorithms may appear to perform satisfactorily, there are still several methodological barriers that can affect the results and increase heterogeneity. Technical parameters like hyperparameter tuning are often kept confidential, resulting in significant statistical heterogeneity. Heterogeneity, which measures the difference in effect size between studies, can arise from several factors like model fine-tuning, hyperparameter selection, and the number of epochs. In addition, data partitioning is arbitrary due to the lack of standard guidelines for utilization. In this study, most included studies had an imbalanced ratio of training and testing datasets, which could lead to poor generalization or even misleading accuracy. It 's essential to consider the generalizability of the studies, as most were developed and validated using Asian populations, with only one study evaluating AI performance in American populations. Evidence has shown that Asians have the highest prevalence of CHD, so more datasets based on other ethnicities are necessary to ensure the study's generalizability (43).

One major issue with deep learning is its black box-like nature, which makes it difficult to understand how it operates and makes decisions. Despite being highly accurate, healthcare workers cannot accept its decisions without proper interpretation. A possible solution to this problem is using interpretable hand-crafted features from clinical information or biosignals that human experts are familiar with and incorporating them into deep learning models to improve their interpretability.

AI has some limitations that should be acknowledged. To improve algorithm performance, a significant amount of training data is required. In addition, the high computational power of AI can lead to over-fitting, where the model is too closely tailored to the training data and cannot adapt to new data.

In summary, artificial intelligence models, especially deep learning techniques, have shown effective results in detecting CHD. However, it is important to carefully consider various factors such as the data acquisition process, characteristics of the data, characteristics of the population being analyzed, weight reduction of the algorithm, working principle, and interpretability of the model to develop a practical medical AI model that can be applied in real-world scenarios.

## Conclusion

While there are some obstacles to using AI models in clinical practice, there is potential for AI to improve CHD diagnosis. However, more extensive studies are necessary to compare AI algorithms with conventional methods and to include a broader range of patients. Once these studies are completed and AI algorithms are validated, they may be helpful in clinical practice, especially in LMICs.

## Data Availability

The raw data supporting the conclusions of this article will be made available by the authors without undue reservation.
