# Supplementary material for: Diagnostic accuracy of artificial intelligence models in detecting congenital heart disease in the second-trimester fetus through prenatal cardiac screening: a systematic review and meta-analysis
Source: Front Cardiovasc Med. 2025 Feb 24;12:1473544. doi: 10.3389/fcvm.2025.1473544 (PMC11891181; doi:10.3389/fcvm.2025.1473544)
Supplement: Supplementary file 1 [file Datasheet1.pdf]

Supplementary Material. Search Strategy

Search concepts and terms

Second trimester fetus

(fetus or fetal or foet* or prenatal or prepart* or (2nd trimester) or (second trimester) or (mid* pregnan*) or (mid* gestation*))
Fetus/ or Pregnancy Trimester, Second/

congenital heart disease

(tetralogy fallot or pulmonary stenosis or congenital heart disease or congenital cardiac disease or congenital heart defect or congenital heart malformation or hypoplastic left heart syndrome)
tetralogy of fallot/ or pulmonary valve stenosis/ or heart defects, congenital/ or fontan procedure/ or univentricular heart/ or hypoplastic left heart syndrome/ or heart septal defects, atrial/ or heart septal defects, ventricular/ or heart septal defects/ or truncus arteriosus/ or ebstein anomaly/ or ebstein anomaly/ or tricuspid atresia/ or transposition of great vessels/ or aortic coarctation/ or cor triatriatum/ or coronary vessel anomalies/

ultrasound screening

(screen* or scan* or structural assessment* or structural survey* or diagnos* or predict* or detect* or ultrasound or ultrasonogra* or sonography or echocardiogra*)
prenatal diagnosis/ or prenatal screening/ or ultrasound/ or ultrasonography/ or echocardiography/ or ultrasonography, prenatal/

Artificial intelligence

(machine learning) or (artificial intelligence) or (deep learning) or (neural network) or (support vector machine) or (random forest)
exp artificial intelligence/ or machine learning/ or deep learning/ or supervised machine learning/ or support vector machine/ or unsupervised machine learning/ or decision theory/ or neural networks, computer/ or diagnosis, computer-assisted/

Accuracy

(accura* or valid* or precision or sensitiv* or specific* or (negative predictive value) or (positive predictive value) or f1 or (receiver operating characteristic) or (area under the curve) or dice or kappa)
ROC Curve/ or Area Under Curve/ or data accuracy/ or "sensitivity and specificity"/ or "predictive value of tests"/

Search results (Finalized on Sept 30th, 2023)

Table … Search results from Pubmed, IEEE Xplore, Scopus, and Cochrane Databases

**Pubmed**		
	(((((fetus or fetal or foet* or prenatal or prepart* or (2nd trimester) or (second trimester) or (mid* pregnan*) or (mid* gestation*)) AND ((tetralogy fallot or pulmonary stenosis or congenital heart disease or congenital cardiac disease or congenital heart defect or congenital heart malformation or hypoplastic left heart syndrome))) AND ((screen* or scan* or structural assessment* or structural survey* or diagnos* or predict* or detect* or ultrasound or ultrasonogra* or sonography or echocardiogra*))) AND ((machine learning) or (artificial intelligence) or (deep learning) or (neural network) or (support vector machine) or (random forest))) AND ((accura* or valid* or precision or sensitiv* or specific* or (negative predictive value) or (positive predictive value) or f1 or (receiver operating characteristic) or (area under the curve) or dice or kappa))	48
**IEEE Xplore**		

	("All Metadata":(fetus or fetal or foet* or prenatal or prepart* or (2nd trimester) or (second trimester) or (mid* pregnan*) or (mid* gestation*))) AND ("All Metadata":(tetralogy fallot or pulmonary stenosis or congenital heart disease or congenital cardiac disease or congenital heart defect or congenital heart malformation or hypoplastic left heart syndrome)) AND ("All Metadata":(screen* or scan* or structural assessment* or structural survey* or diagnos* or predict* or detect* or ultrasound or ultrasonogra* or sonography or echocardiogra*)) AND ("All Metadata":(machine learning) or (artificial intelligence) or (deep learning) or (neural network) or (support vector machine) or (random forest)) AND ("All Metadata":(accura* or valid* or precision or sensitiv* or specific* or (negative predictive value) or (positive predictive value) or f1 or (receiver operating characteristic) or (area under the curve) or dice or kappa))	0
**Scopus**		
	(ALL ((fetus OR fetal OR foet* OR prenatal OR prepart* OR (2nd AND trimester) OR (second AND trimester) OR (mid* AND pregnan*) OR (mid* AND gestation*))) AND ALL ((tetralogy AND fallot OR pulmonary AND stenosis OR congenital AND heart AND disease OR congenital AND cardiac AND disease OR congenital AND heart AND defect OR congenital AND heart AND malformation OR hypoplastic AND left AND heart AND syndrome)) AND ALL ((screen* OR scan* OR structural AND assessment* OR structural AND survey* OR diagnos* OR predict* OR detect* OR ultrasound OR ultrasonogra* OR sonography OR echocardiogra*)) AND ALL ((machine AND learning) OR (artificial AND intelligence) OR (deep AND learning) OR (neural AND network) OR (support AND vector AND machine) OR (random AND forest)) AND ALL ((accura* OR valid* OR precision OR sensitiv* OR specific* OR (negative AND predictive AND value) OR (positive AND predictive AND value) OR f1 OR (receiver AND operating AND characteristic) OR (area AND under AND the AND curve) OR dice OR kappa)))	177
**Cochrane**		
1	((accura* or valid* or precision or sensitiv* or specific* or (negative predictive value) or (positive predictive value) or f1 or (receiver operating characteristic) or (area under the curve) or dice or kappa))	338711
2	MeSH descriptor: [ROC Curve] explode all trees	1691
3	MeSH descriptor: [Artificial Intelligence] explode all trees	2929
4	MeSH descriptor: [Heart Defects, Congenital] explode all trees	2881
5	MeSH descriptor: [Ultrasonography, Prenatal] explode all trees	699
6	MeSH descriptor: [Pregnancy Trimester, Second] explode all trees	774
7	((machine learning) or (artificial intelligence) or (deep learning) or (neural network) or (support vector machine) or (random forest)):ti,ab,kw	7322
8	((screen* or scan* or structural assessment* or structural survey* or diagnos* or predict* or detect*) (ultrasound or ultrasonogra* or sonography or echocardiogra*)):ti,ab,kw	35105
9	((tetralogy fallot or pulmonary stenosis or congenital heart disease or congenital cardiac disease or congenital heart defect or congenital heart malformation or hypoplastic left heart syndrome))	4325
10	((fetus or fetal or foet* or prenatal or prepart* or (2nd trimester) or (second trimester) or (mid* pregnan*) or (mid* gestation*)))	36551
11	MeSH descriptor: [Sensitivity and Specificity] explode all trees	19944
12	MeSH descriptor: [Data Accuracy] explode all trees	118
13	MeSH descriptor: [Fetus] explode all trees	2470
14	(ultrasound* or ultra-sound or ultrasonogra* or ultra-sonogra* or sonogra* or echocardiogra*)	73114
15	(#1 OR #2 OR #11 OR #12) AND (#3 OR #7) AND (#4 OR #9) AND (#5 OR #8 OR #14) AND (#6 OR #13 OR #10)	5

Table … Search results for Medline, Global Health, and Embase Databases

	Keywords	Medline	Global Health	Embase
1	(fetus or fetal or foet* or prenatal or prepart* or (2nd trimester) or (second trimester) or (mid* pregnan*) or (mid* gestation*))	596145	71524	827605
2	Fetus/ or Pregnancy Trimester, Second/	98247	18205	261303
3	(tetralogy fallot or pulmonary stenosis or congenital heart disease or congenital cardiac disease or congenital heart defect or congenital heart malformation or hypoplastic left heart syndrome)	45251	2340	122822
4	tetralogy of fallot/ or pulmonary valve stenosis/ or heart defects, congenital/ or fontan procedure/ or univentricular heart/ or hypoplastic left heart syndrome/ or heart septal defects, atrial/ or heart septal defects, ventricular/ or heart septal defects/ or truncus arteriosus/ or ebstein anomaly/ or ebstein anomaly/ or tricuspid atresia/ or transposition of great vessels/ or aortic coarctation/ or cor triatriatum/ or coronary vessel anomalies/	120898	103	107486
5	(screen* or scan* or structural assessment* or structural survey* or diagnos* or predict* or detect* or ultrasound or ultrasonogra* or sonography or echocardiogra*)	10489569	1342028	14534265
6	prenatal diagnosis/ or prenatal screening/ or ultrasound/ or ultrasonography/ or echocardiography/ or ultrasonography, prenatal/	363835	21447	852944
7	(machine learning) or (artificial intelligence) or (deep learning) or (neural network) or (support vector machine) or (random forest)	243646	12802	324599
8	exp artificial intelligence/ or machine learning/ or deep learning/ or supervised machine learning/ or support vector machine/ or unsupervised machine learning/ or decision theory/ or neural networks, computer/ or diagnosis, computer-assisted/	200180	3058	293071
9	(accura* or valid* or precision or sensitiv* or specific* or (negative predictive value) or (positive predictive value) or f1 or (receiver operating characteristic) or (area under the curve) or dice or kappa)	7138802	871264	9176347
10	ROC Curve/ or Area Under Curve/ or data accuracy/ or "sensitivity and specificity"/ or "predictive value of tests"/	636298	0	790688
11	1 or 2	599754	71524	827605
12	3 or 4	136278	2393	176439
13	5 or 6	10489569	1342028	14561168
14	7 or 8	323363	12802	377640
15	9 or 10	7252764	871264	9216947
16	11 and 12 and 13 and 14 and 15	31	0	95
